# Genetic Diversity of *Bartonella* spp. in Cave-Dwelling Bats and Bat Flies, Costa Rica, 2018

**DOI:** 10.3201/eid2802.211686

**Published:** 2022-02

**Authors:** Miranda M. Mitchell, Amanda Vicente-Santos, Bernal Rodríguez-Herrera, Eugenia Corrales-Aguilar, Thomas R. Gillespie

**Affiliations:** Emory University, Atlanta, Georgia, USA (M.M. Mitchell, A. Vicente-Santos, T.R. Gillespie);; University of Costa Rica, San José, Costa Rica (B. Rodríguez-Herrera, E. Corrales-Aguilar)

**Keywords:** Bartonella spp., bats, bat flies, Costa Rica, genetic diversity, bacteria

## Abstract

To determine *Bartonella* spp. dynamics, we sampled bats and bat flies across 15 roosts in Costa Rica. PCR indicated prevalence of 10.7% in bats and 29.0% in ectoparasite pools. Phylogenetic analysis of 8 sequences from bats and 5 from bat fly pools revealed 11 distinct genetic variants, including 2 potentially new genotypes.

*Bartonella*, the causative agent of bartonellosis, is a genus of gram-negative bacteria. Bartonellosis causes a range of symptoms from severe to life-threatening (e.g., endocarditis and meningitis). Clinical syndromes from *Bartonella* infections include trench fever (*B. quintana*), cat scratch disease (*B. henselae*), and Carrion’s disease (*B. bacilliformis*) (*1*). Bats (Order Chiroptera) and their blood-feeding ectoparasitic bat flies (Superfamily Hippoboscoidea) host a diversity of *Bartonella* species, awakening interest in their potential role as natural reservoirs for this pathogen (*2*,*3*). To learn more about this interplay, we examined the genetic diversity and geographic sharing of *Bartonella* spp. in diverse assemblages of bats and bat flies across Costa Rica.

In 2018, we nonlethally sampled 321 bats (18 species) by using hand nets, mist nets, and harp traps across 15 roosts throughout Costa Rica (Appendix Figure). We took blood samples from 252 bats (16 species) and collected 114 ectoparasites from 48 bats, following Emory University Institutional Animal Care and Use Committee protocol (DAR-4000049-ENTRPR-N) and with the approval of the National System of Conservation Areas (SINAC-Costa Rica) (research permit nos. R-SINAC-PNI-ACAHN-016–2018, M-P-SINAC-PNI-ACAT-035–2018, SINAC-ACC-PI-R-068–2018, R-SINAC-ACG-PI-030–2018, R-SINAC-PNI-ACLAC-044–2018, SINAC-ACOPAC-D-RES-063–2018, INV-ACOSA-046–18, ACT-OR-DR-066–18). We taxonomically identified the bats and bat flies (*4*–*6*), pooled the bat flies (1–8 bat flies/pool) by individual bat host and bat fly species (62 pools) and extracted DNA from bat blood and ectoparasite pooled samples. We screened extracted DNA for *Bartonella* spp. by amplifying a 770-bp portion of the partial citrate synthase gene (*gltA*) (*7*) and using *B. doshiae* as a positive control (provided by M. Kosoy, M. Rosales Rizzo, Centers for Disease Control and Prevention). Samples positive by PCR were sequenced for confirmation.

To create global phylogenies, we trimmed obtained consensus sequences to 768 bp and aligned them to 45 genetic sequences: 28 from known *Bartonella* species, 12 *Bartonella* sequences from bats and bat flies in Costa Rica (*8*), 4 sequences from bats in Guatemala (*9*), and 1 sequence from Mexico (*3*). We used *B. tamiae* and *Brucella melitensis* as outgroups to root the tree. We created the alignment by using the multiple alignment program MAFFT (https://mafft.cbrc.jp/alignment/software), manually checked in MEGA X (https://www.megasoftware.net), and further refined with alignment refinement tool Gblocks version 0.91b (http://molevol.cmima.csic.es/castresana/Gblocks/Gblocks_documentation.html). We constructed the global phylogenetic tree by using Bayesian Markov chain Monte Carlo analyses (MrBayes 2.2.4, https://www.geneious.com) with 1 million generations and a burn-in fraction of 25% and determined the parameters for the nucleotide changes (MEGA X).

*Bartonella* prevalence from all samples, determined by PCR, was 14.3% (45/314), 10.7% (27/252) for bats and 29.0% (18/62) for ectoparasite pools (Table). *Bartonella* seems to be widespread and diverse in bats and bat flies in Costa Rica, where 6 of the 16 bat species and 9 of the 23 bat fly species were positive for the bacterium. Because of sequence quality, we included only 8 *Bartonella* sequences from bats and 6 from bat fly pools in phylogenetic analyses, which revealed 11 genetic variants, including 2 potentially new genotypes (93.2% similarity value; Figure; Appendix Table). These 11 genetic variants clustered into 9 clades of 96.0%–99.2% similarity.

**Table:** Prevalence of *Bartonella* spp. in bats and bat flies sampled from roost sites, Costa Rica, 2018

Species	No. positive/no. sampled
Bats	
* Artibeus jamaicensis*	0/1
* Balantiopteryx plicata*	0/4
* Carollia perspicillata*	19/79
* Desmodus rotundus*	1/25
* Diphylla ecaudata*	0/1
* Glossophaga commisarisi*	0/12
* Glossophaga soricina*	0/10
* Lonchophylla robusta*	1/25
* Lonchorhina aurita*	0/13
* Macrophyllum macrophyllum*	1/1
* Phyllostomus hastatus*	0/4
* Pteronotus gymnonotus*	3/11
* Pteronotus mesoamericanus*	2/56
* Pteropteryx kappleri*	0/1
* Tonatia saurophilia*	0/1
* Trachops cirrhosis*	0/8
Total	27/252

Bat flies	
* Aspidoptera phyllostomasis*	0/1
* Exastinion clovisi*	2/2
* Megistopoda aranea*	1/4
* Speiseria ambigua*	0/1
* Strebla carolliae*	0/1
* Strebla diaemi*	0/1
*Strebla galindoi*†	1/2
* Strebla guajiro*	0/1
*Strebla hertigi*†	1/1
* Strebla mirabilis*	0/1
*Strebla vespertilionis*†	2/2
* Trichobius cecus*	0/3
* Trichobius dugesiodes*	0/2
* Trichobius dunni*	0/1
* Trichobius furmani*	0/1
* Trichobius galei*	0/3
* Trichobius johnsonae*	1/3
* Trichobius keenani*	0/1
*Trichobius pallidus*†	7/22
* Trichobius perspicillata*	0/1
*Trichobius sparsus*†	2/3
*Trichobius uniformis*†	1/2
* Trichobius yunkeri*	0/3
Total	18/62

*For bat flies, no. sampled indicates no. sampled pools. †Newly described species with *Bartonella*.

**Figure:**
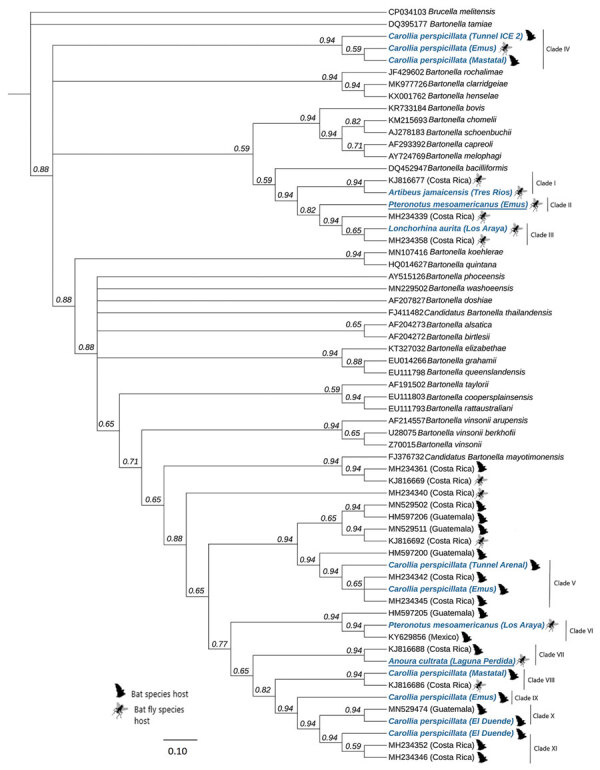
Phylogenetic tree of 768 bp partial *gltA* gene of *Bartonella* variants found in study of *Bartonella* spp. in bats and bat flies sampled from roost sites, Costa Rica, 2018 (blue), compared with globally named species and other variants found in bats and bat flies in Central America and Mexico. Each sequence is labeled with its GenBank accession number, the organism on which it was detected, and the country of origin. For species in this study, we included the specific site (accession numbers in Appendix Table). Underlining indicates the potential newly described genotypes. We constructed the global phylogenetic tree by using Bayesian Markov chain Monte Carlo (MrBayes 2.2.4, https://www.geneious.com), with 1 million generations and a burn-in fraction of 25%. We determined the parameters for the nucleotide changes by using MEGA X (https://mafft.cbrc.jp/alignment/software). Inner node labels identify consensus support. Scale bar indicates nucleotide substitutions/site (%).

Our results suggest that within Costa Rica variants are shared between bats and their flies in different parts of the country and in different years. For example, *Bartonella* sequences from Emus Cave (GenBank accession no. MW115627) and Túnel Arenal (GenBank accession no. MW115628) at opposite ends of the country (clade V; Appendix Figure) clustered together with sequences from a study conducted in Costa Rica in 2015 (*8*). In addition, *Bartonella* sequences from our study clustered with previously identified sequences from bats and bat flies from Guatemala (*9*) and Mexico (*3*), suggesting wide geographic distribution.

We also found a high level of diversity of *Bartonella* variants within caves and species (Figure). For example, *Bartonella* sequences from different bats (of same and different species) in Emus Cave clustered in 4 distinct clades. In addition, *Carollia perspicillata* bats, the most sampled species in our study, carried *Bartonella* with sequences from 6 distinct clades. This finding suggests that >1 *Bartonella* strain is circulating within bat species, even within the same cave.

When assessing spillover risk to humans and domestic animals, we found that the *Bartonella* sequences we detected did not cluster with *Bartonella* species known to cause infection in humans and other animals and did not significantly overlap with sequences from any globally identified species (Figure). To fully assess potential for *Bartonella* spillover from bat and bat fly species to other animals and humans, further analyses should be conducted.

In conclusion, we found *Bartonella* species to be diverse, prevalent, and potentially widely shared among species of bats and bat flies in Costa Rica and Mesoamerica. We expanded existing scientific knowledge on the prevalence and diversity of *Bartonella* in bats and bat flies in Costa Rica by including species that were not previously tested and described as positive by PCR for these bacteria. We also described 2 new *Bartonella* genotypes through phylogenetic analysis. Information about the dynamics of *Bartonella* in its natural hosts can be used to predict and avert further *Bartonella* emergence.

Appendix Roost sites and number of bats sampled per roost for *Bartonella* spp. and GenBank accession numbers and distribution of 11 genetic variants of *Bartonella* spp. in Costa Rica, 2018.
