# Supplementary material for: Genetic Diversity of Bartonella spp. in Cave-Dwelling Bats and Bat Flies, Costa Rica, 2018
Source: Emerg Infect Dis. 2022 Feb;28(2):488–91. doi: 10.3201/eid2802.211686 (PMC8798684; doi:10.3201/eid2802.211686)
Supplement: Appendix — Roost sites and number of bats sampled per roost for Bartonella spp. and GenBank accession numbers and distribution of 11 genetic variants of Bartonella spp. in Costa Rica, 2018. [file 21-1686-Techapp-s1.pdf]

Article DOI: https://doi.org/10.3201/eid2802.211686

Genetic Diversity of *Bartonella* spp. in Cave-Dwelling Bats and Bat Flies, Costa Rica, 2018

Appendix

Appendix Table. GenBank accession numbers and distribution of 11 genetic variants of *Bartonella* spp. found in bats and bat flies sampled from roost sites in Costa Rica

Accession no.	Roost	Host species	Clade
MW115627	Emus	*Carollia perspicillata*	IX
MW115628	Túnel Arenal	*Carollia perspicillata*	IX
MW115629	Emus	*Carollia perspicillata*	VII
MW115630	El Duende	*Carollia perspicillata*	VII
MW115631	El Duende	*Carollia perspicillata*	VII
MW115632	Mastatal	*Carollia perspicillata*	VIII
MW115624	Emus	*Carollia perspicillata*	IV
MW115625	Túnel ICE 2	*Carollia perspicillata*	IV
MW115626	Mastatal	*Trichobius pallidus* on *C. perspicillata*	IV
MW115635	Tres Ríos	*Megistopoda aranea* on *Artibeus jamaicensis**	I
MW115637	Los Araya	*Trichobius lionycterdis* on *Lonchorhina aurita*	III
MW115636†	Emus	*Trichobius pallidus* on *Pteronotus mesoamericanus*	II
MW115633	Los Araya	*Trichobius pallidus* on *Pteronotus mesoamericanus*	V
MW115634†	Laguna Perdida	*Exastinion clovisi* on *Anoura cultrata**	VI

*Blood sample not collected.
†Newly described genotype with a similarity value of 93.2%.

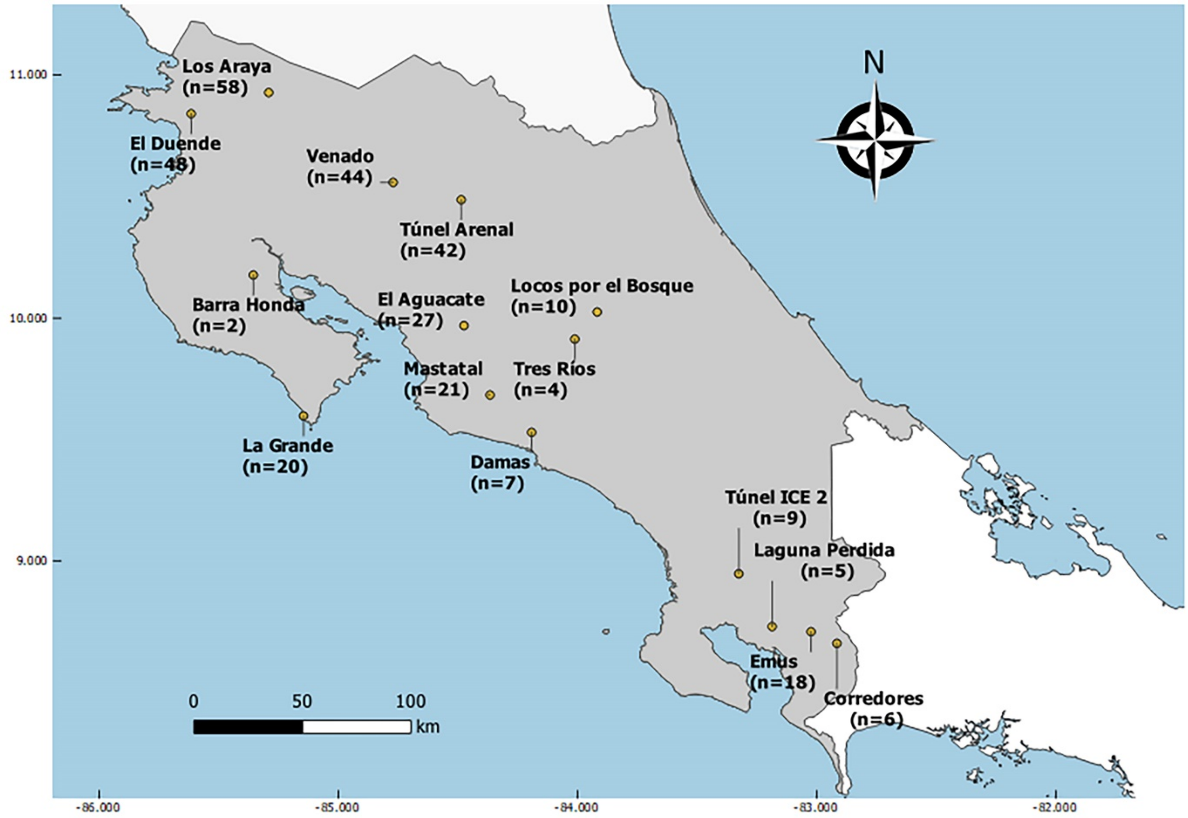

Appendix Figure. Roost sites and number of bats sampled per roost for *Bartonella* spp. in Costa Rica. Map was created using qGIS 3.13.8 software (https://www.qgis.org/en/site).
